# Supplementary material for: Expression and Retention of Thymidine Phosphorylase in Cultured Reticulocytes as a Novel Treatment for MNGIE
Source: Mol Ther Methods Clin Dev. 2020 Apr 1;17:822–30. doi: 10.1016/j.omtm.2020.03.029 (PMC7191122; doi:10.1016/j.omtm.2020.03.029)
Supplement: Document S1. Figures S1 and S2 [file mmc1.pdf]

OMTM, Volume *17*

Supplemental Information

Expression and Retention of Thymidine Phosphorylase in Cultured Reticulocytes as a Novel Treatment for MNGIE

Marjolein Meinders, Debbie Shoemark, Johannes G.G. Dobbe, Geert J. Streekstra, Jan Frayne, and Ashley M. Toye

Supplemental Figure 1

A

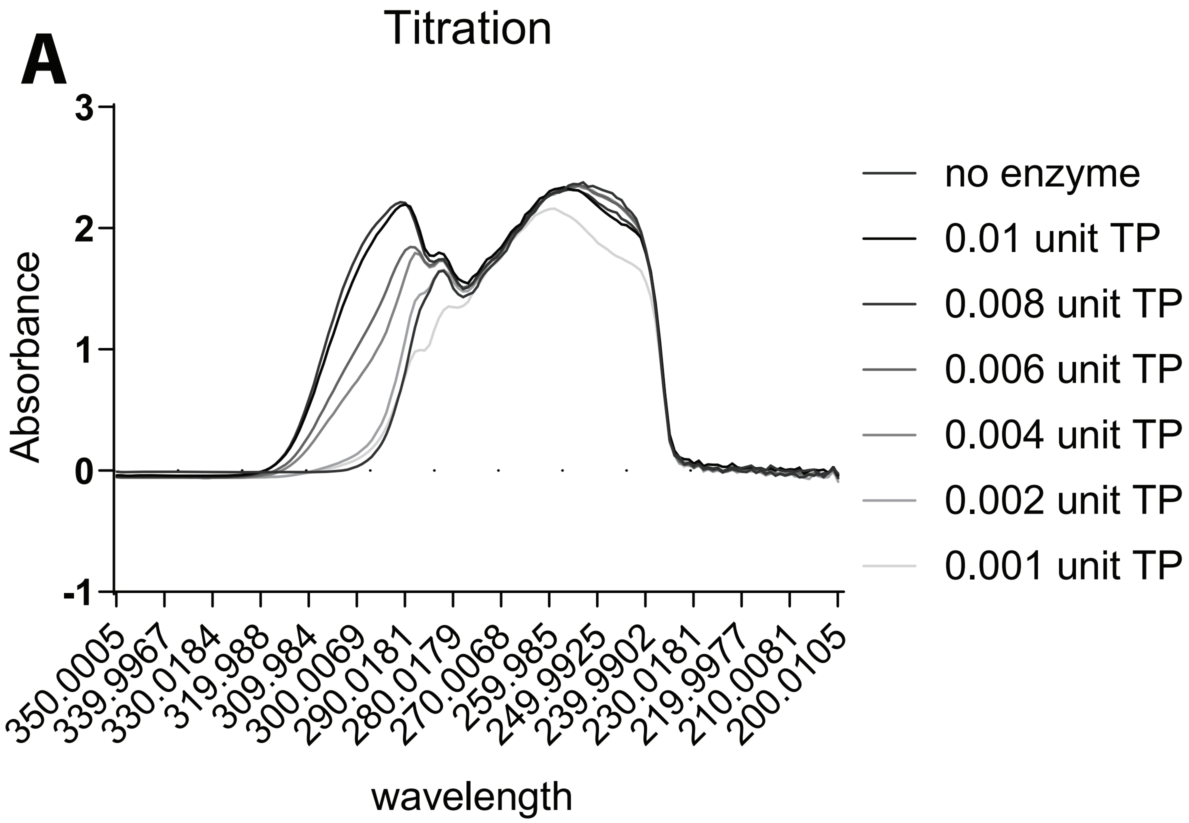

B

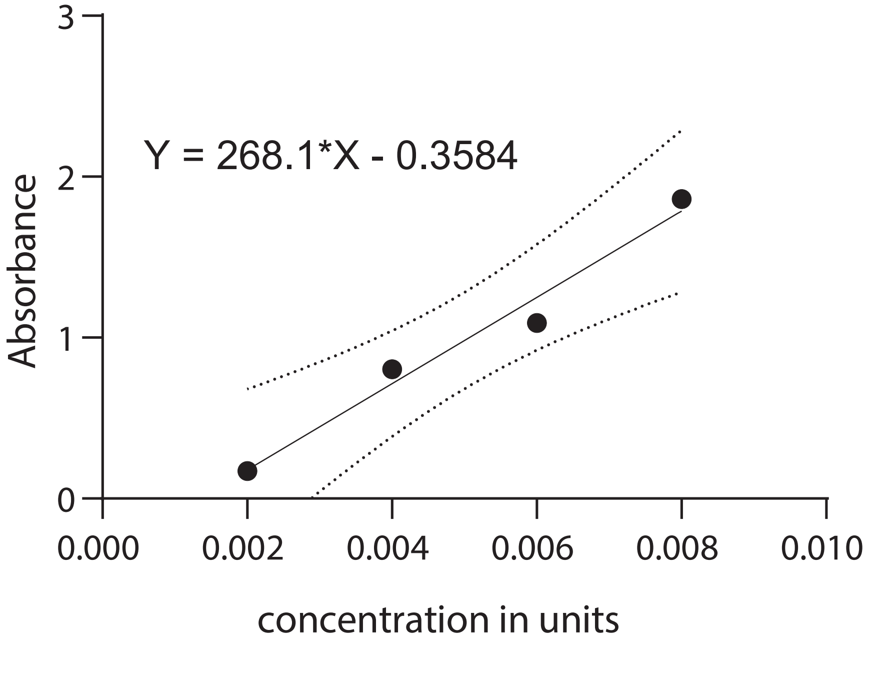

Supplemental Figure 2

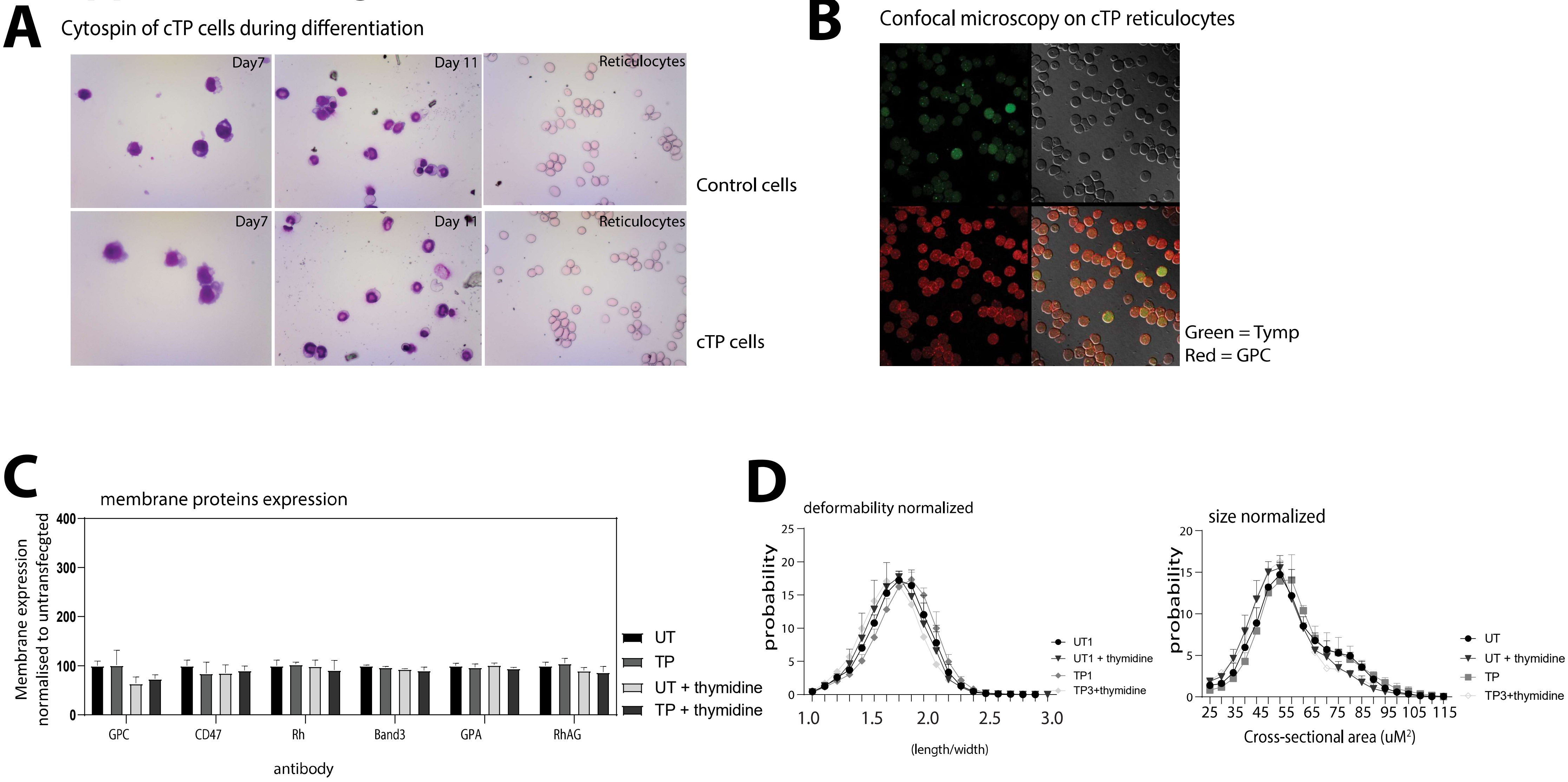

Supplementary Figure 1. TP activity assay data acquisition and calculation

For each individual experiment a standard curve was done to calculate the concentration of TP in our cells in units/cells. An example of the calculation is shown in Supplemental Figure 1. (A) shows the reduction of thymidine upon incubation with a known amount of enzyme after 1-hour incubation at 37°C. (B) shows the absolute absorbance values at wavelength of 299nm. We are able to fit a regression line through the data points from which we can calculate the formula y=268.1X-0.3584, whereby X is the concentration in the cell and Y the absorbance. The concentration of TP is in units, which is defined as 1U (µmol/min) is the amount of the enzyme that catalyses the conversion of one micromole of thymidine per minute. This is an example calculation. For each individual experiment, a regression line was plotted, and the concentration was calculated.

Supplementary Figure 2. Additional analysis of cTP reticulocytes

(A) Representative images illustrating cell morphology in UT and cTP cells during differentiation. At indicated times of cell culture, $1x10^4$ cells were cytospun and stained with a may-grunwald giemsa stain. (B) Reticulocytes from cTP were fixed and labelled with a TP (green) and a GPC (red) antibody and imaged using confocal microscopy which confirmed the cytosolic localization of TP in the reticulocytes. (C) Membrane protein expression was measured using flow cytometry on reticulocytes of GPC, CD47, Rh, Band3, GPA, RhAG from untransduced (UT) or untransduced cultured with 0.5mM thymidine, cTP, and cTP cells cultured with thymidine (N=3 ± SEM). (D) Automated Rheoscope and Cell Analyzer (ARCA) measurements of deformability and size for UT (untransduced cells) and cTP reticulocytes cultured in the presence of 0.5mM thymidine (n=3 ± SEM).
