# Supplementary material for: Expression and Retention of Thymidine Phosphorylase in Cultured Reticulocytes as a Novel Treatment for MNGIE
Source: Mol Ther Methods Clin Dev. 2020 Apr 1;17:822–30. doi: 10.1016/j.omtm.2020.03.029 (PMC7191122; doi:10.1016/j.omtm.2020.03.029)
Supplement: Document S2. Article plus Supplemental Information [file mmc2.pdf]

Molecular Therapy
Methods & Clinical Development
Original Article

Expression and Retention of Thymidine Phosphorylase in Cultured Reticulocytes as a Novel Treatment for MNGIE

Marjolein Meinders,[1,2,3] Debbie Shoemark,[1,2] Johannes G.G. Dobbe,[4] Geert J. Streekstra,[4] Jan Frayne,[2,5] and Ashley M. Toye[1,2,3,5]

[1]Bristol Synthetic Biology Centre (BrisSynBio), University of Bristol, Bristol BS8 1TD, UK; [2]School of Biochemistry, University of Bristol, Biomedical Sciences Building, University Walk, Bristol BS8 1TD, UK; [3]NIHR Blood and Transplant Research Unit in Red Blood Cell Products, University of Bristol, Bristol BS8 1TD, UK; [4]Department of Biomedical Engineering and Physics, Amsterdam UMC, University of Amsterdam, Meibergdreef 9, 1105 AZ Amsterdam, the Netherlands; [5]Bristol Institute for Transfusion Sciences, National Health Service Blood and Transplant (NHSBT), Filton, Bristol BS34 7QH, UK

Mitochondrial neurogastrointestinal encephalomyopathy (MNGIE) is a rare autosomal metabolic disorder caused by thymidine phosphorylase (TP) deficiency. Successful therapeutic interventions for this disease rely on a means for efficient and long-lasting circulation of the TP enzyme. In this study we exploit lentiviral transduction of hematopoietic stem cells and an erythroid cell line (BEL-A) to generate reticulocytes that contain active TP. Significant loss of overexpressed TP during erythroid differentiation can be reduced by addition of the ubiquitination inhibitor MG132. However, the ubiquitination sites are located in the substrate binding site in human TP, and their removal abolished enzyme activity. Examination of the TP structure and mechanism suggested that these sites are only exposed in the absence of substrate. We show that supplementation of culture media with thymidine during differentiation reduces enzyme degradation, doubling the amount of TP retained in reticulocytes. This study provides proof of principle that therapeutic reticulocytes expressing TP can be generated *in vitro* and that ubiquitin-mediated degradation can be subverted through masking ubiquitination sites to ensure retention of human TP in reticulocytes following erythroid differentiation.

INTRODUCTION

Mitochondrial neurogastrointestinal encephalomyopathy (MNGIE) is an autosomal recessive metabolic disorder[1] that usually manifests during early teens, with an average life expectancy of 37.5 years.[2,3] The disease is very rare, with an estimated occurrence in the population of 1:1,000,000 but this may be an underestimate[4] due to the varied clinical presentation, which includes severe gastrointestinal dysmotility, cachexia, peripheral neuropathy, diffuse leukoencephalopathy, and mitochondrial abnormalities.[3]

MNGIE is caused by mutations in the *TYMP* gene, which encodes the thymidine phosphorylase (TP) enzyme. TP catalyzes the phosphorolysis of thymidine (dThd) and deoxyuridine (dUrd) to thymine or uridine, and 2-deoxy ribose 1-phosphate (2DR1P) in the cytosol. Here it plays a key role in pyrimidine salvage, recovering nucleosides after DNA/RNA degradation.[5] Homozygous or compound heterozygous mutations in the *TYMP* gene cause a drastic reduction in protein expression or activity, which results in thymidine accumulation, and subsequently leads to an imbalanced intramitochondrial deoxynucleotide pool.[4,6–8] This is thought to destabilize mitochondrial DNA by affecting mitochondrial DNA repair and replication, resulting in the broad variety of symptoms.[9]

Although significant progress in the understanding on the molecular basis of the MNGIE has been made, we still lack an effective treatment. Currently, treatment is largely based on patient symptom management, which include nutritional supplements, prevention of infections, and pain relief. Research has focused on developing treatments to remove metabolites using hemodialysis, hematopoietic stem cell transplantation (HSCT), and TP enzyme replacement therapy.[4] Although hemodialysis is beneficial, the effect is transient, and dialysis is required every 3 h.[8] HSCT can restore expression of TP and improve the biochemical parameters, but transplantation has inherent risks, and achieving a suitable donor match can be challenging.[10] A different method of increasing TP activity is the use of enzyme replacement therapy in platelets and red blood cells. Platelets naturally express high levels of TP, and platelet transfusion corrected the nucleoside imbalance in blood plasma. However, the improvement was temporary and multiple platelet transfusions per week are necessary for long-term improvements.[4]

The most promising approach for enzyme replacement is the use of erythrocyte encapsulated TP (EETP). Erythrocytes do not normally express TP, but hypotonic hemolysis and isotonic resealing[11] can be used to encapsulate *Escherichia coli* TP in autologous red blood

Received 10 March 2020; accepted 25 March 2020;
https://doi.org/10.1016/j.omtm.2020.03.029.

Correspondence: Ashley Mark Toye, Bristol Synthetic Biology Centre (BrisSynBio), University of Bristol, Bristol BS8 1TD, UK.
E-mail: ash.m.toye@bristol.ac.uk

 © 2020 The Authors.
This is an open access article under the CC BY license (http://creativecommons.org/licenses/by/4.0/).

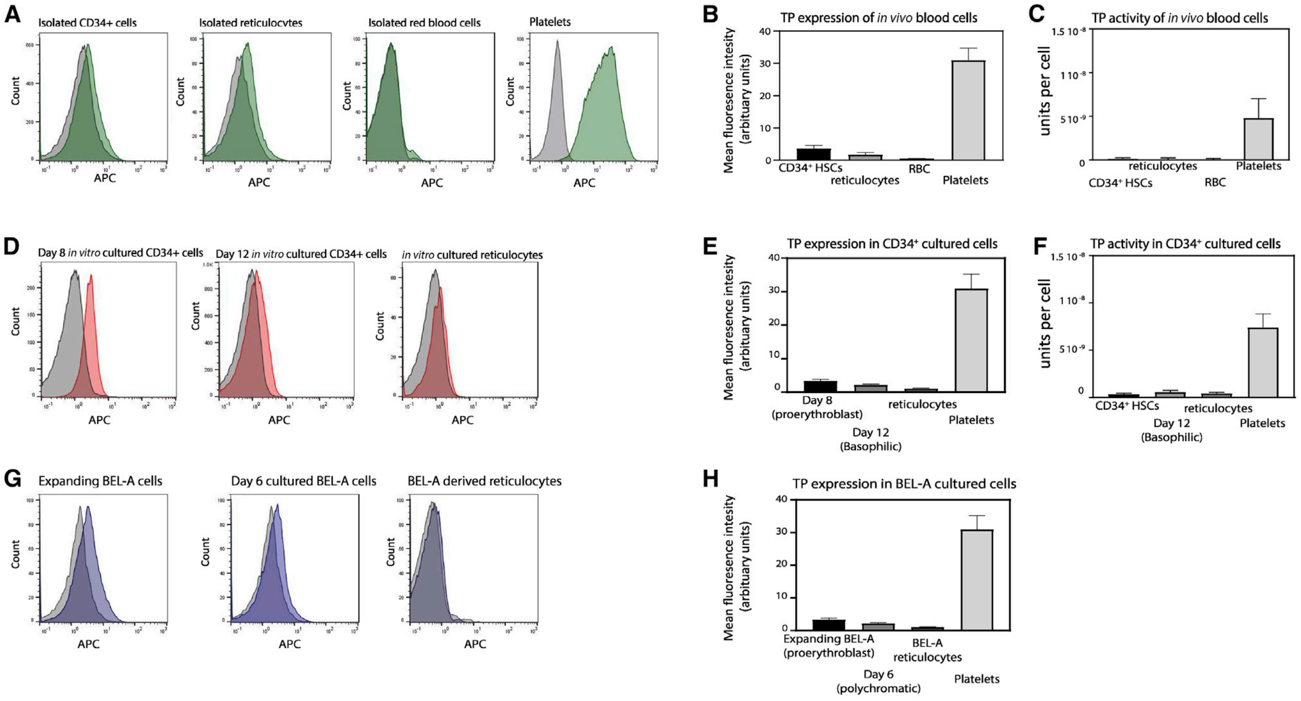

Figure 1. Endogenous TP Expression and Activity

(A, D, and G) Representative flow cytometry histograms show the expression levels of TP in 1×10^5 fixed and permeabilized cells in (A) isolated $CD34^+$ cells, isolated native reticulocytes, red blood cells (RBCs), and isolated platelets, (D) *in vitro*-cultured erythroblasts and reticulocytes, and (G) in expanded BEL-A and differentiated BEL-A cells. (B, E, and H) The average measurements are shown for 1×10^5 fixed and permeabilized cells by flow cytometry in mean fluorescence intensity (arbitrary units) for (B) isolated blood cells, (E) *in vitro*-cultured erythroblasts and reticulocytes, and (H) expanding BEL-A (proerythroblasts), differentiating BEL-A on day 6 (polychromatic), and BEL-A-derived reticulocytes. Data are represented as mean ± SEM (N = 6). (C and F) TP activity was measured using 1×10^6 lysed cells by a spectrophotometry-based assay (see Materials and Methods, and Figure S1 for example titration curve). 1 U (μmol/min) is defined as the amount of the enzyme that catalyzes the conversion of μmol of thymidine per minute. (C) TP activity in isolated $CD34^+$ cells, reticulocytes, and red blood cells, and, as a positive control, TP activity was measured in 1×10^7 platelets (N = 3 ± SEM). (F) TP activity was measured in 1×10^6 *in vitro*-cultured red blood cells.

cells.[12] This has been successfully used in the clinic, achieving prolonged cessation of the MNGIE clinical phenotype by reducing plasma nucleoside levels.[13,14] Although this method is promising, the methodology of encapsulation within erythrocytes using hypotonic lysis can compromise the lifespan of the erythrocytes, and patients can develop antibodies against the bacterial enzyme.[15] Recently, progress has been made in the *ex vivo* culture of reticulocytes from $CD34^+$ stem cells *in vitro*, with more than 10 mL of leukofiltered packed reticulocytes produced using good manufacturing practice (GMP).[16] This, and the recent development of an adult phenotype erythroid cell line (BEL-A),[17] led us to investigate the possibility of using genetically modified reticulocytes as vehicles for TP transport as a novel therapy for MNGIE.

RESULTS

Endogenous TP Expression in Erythroid Progenitors, Reticulocytes, and Erythrocytes

Before exploring the possibility of genetically modifying reticulocytes to express TP, we first confirmed the baseline endogenous expression and TP activity levels in isolated hematopoietic $CD34^+$ stem cells, standard donor-derived reticulocytes, and erythrocytes. Expression was assessed by flow cytometry on fixed and permeabilized cells, and TP activity was determined on cell lysates using a spectrophotometer-based assay whereby the difference in absorbance levels of thymine, in the presence of TP, after 60 min at 37°C was measured (Figure S1).[18] As expected, both assays confirmed a low endogenous expression and activity of TP in $CD34^+$ hematopoietic stem cells and native peripheral blood reticulocytes and no TP expression or activity in red blood cells (Figures 1A–1C). Freshly isolated platelets from peripheral blood were included as a positive control (Figures 1B and 1C).

Endogenous TP expression and activity in erythroid cells differentiated *in vitro* from $CD34^+$ hematopoietic stem cells was examined. The different stages of erythroid maturation in our *in vitro* culturing system has been reported previously.[19] Hereafter, we refer to the days in culture and their approximate stage of differentiation in parentheses based on this knowledge. Figures 1D–1F show that the expression of endogenous TP and activity in cultured day 8 (proerythroblast) and day 12 (polychromatic erythroblast) is low. The expression and activity of filtered *in vitro*-cultured reticulocytes is also comparable to donor-isolated reticulocytes. We next tested endogenous TP

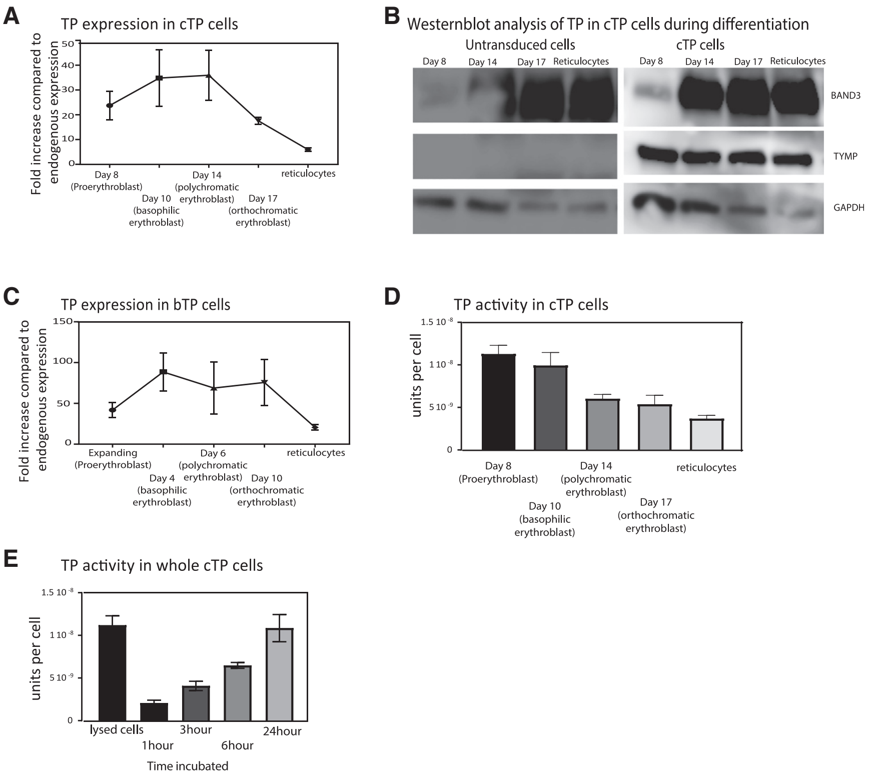

Figure 2. Lentiviral Overexpression of Human TP in Cultured Reticulocytes

(A and C) CD34[+] hematopoietic stem cells and expanding BEL-A cells were transduced using lentivirus with TP cDNA generating cTP (A) and bTP (C) cells and subsequently differentiated into reticulocytes. TP expression was assessed at different time points during differentiation by flow cytometry, where at each indicated time point 1×10^5 cells were fixed and permeabilized and subsequently labeled with a TP antibody. Expression is normalized to endogenous expression of TP at the proerythroblast stage (N = 6 ± SEM). (B) Expression by western blotting of TP, Band3, and GAPDH during differentiation. Equal cell numbers were loaded (1×10^6 cells per lane). (D) TP activity was measured in 1×10^6 cTP cells using the spectrophotometry-based assay at the indicated time point during differentiation (N = 6). (E) Time course of TP activity in whole cells using the spectrophotometry-based assay. Decrease in thymidine concentration was measured in the supernatant of 1×10^6 cells at the indicated time points (N = 3).

expression in an expanding erythroid cell line, BEL-A, which has the capacity to differentiate into reticulocytes, which are comparable to *in vitro*-cultured reticulocytes.[17] The advantage of also using BEL-A cells is that these provide a sustainable source of cells that can be genetically modified and stored frozen with the modification maintained indefinitely, whereas CD34[+]-derived cultures are finite and need to be reinitiated each time. Expanding BEL-A cells (which are comparable to proerythroblast erythroblasts) have a low endogenous TP expression (Figures 1G and 1H), and the BEL-A-derived reticulocytes exhibit a very low TP expression (Figure 1H), comparable to CD34[+]-derived cultured reticulocytes (Figure 1E) and *in vivo*-isolated reticulocytes (Figure 1B).

Exogenous Overexpression of TP in *In Vitro* CD34[+] Reticulocytes and BEL-A-Derived Reticulocytes Using Lentivirus

Cultured erythroid progenitors expressing TP (cTP) and expanding BEL-A cells expressing TP (bTP) were created by stably transducing the cells with lentivirus-expressing TP cDNA. Subclones were created from the polyclonal bTP population by blind single cell sorting using fluorescence-activated cell sorting (FACS). Day 7 cTP (proerythroblast) cells and expanding bTP (proerythroblast) cells exhibited a 25- and 45-fold increase, respectively, in TP enzyme expression by flow cytometry compared to endogenous expression of TP in untransduced (UT) proerythroblast cells (Figures 2A and 2B).

The cTP cells were differentiated and TP expression was measured at the indicated days by flow cytometry (Figure 2A) and by western blot (Figure 2B). bTP expression was measured during differentiation at day 4 (basophilic erythrocytes), day 6 (polychromatic erythrocytes), and day 10 (orthochromatic erythrocytes) and in reticulocytes (see Figure 2C). Although TP expression in cTP and bTP reticulocytes is observed to be 6- and 12-fold increased relative to UT proerythroblast levels, a striking decline of expression is observed during terminal differentiation.

The activity assay confirmed the presence of active enzyme in cTP proerythroblasts with an approximate concentration of 1.2×10^{-8} U/cell (Figure 2D). Subsequently, samples were taken during differentiation at the indicated time points, and activity was measured. We observed an approximately 3-fold reduction of activity in cTP-filtered reticulocytes compared to cTP proerythroblasts, which have a final concentration of 4.4×10^{-9} U/cell. A 3-fold reduced expression was also observed using flow cytometry between cTP proerythroblasts and cTP reticulocytes.

To test whether thymidine and thymine are able to cross the intact reticulocyte cell membrane, and whether TP is active in the intracellular environment of reticulocytes, an activity assay was performed on whole cells. Thymidine was added to the media of 1×10^6 cells at 37°C, and subsequently the concentration of thymine was measured in the supernatant after 1, 3, 6, and 24 h of incubation. Lysed cTP reticulocytes were used as a control, which were incubated with thymidine for 1 h (Figure 2E). As expected, the increase in thymine production is slower in whole-cell supernatants, compared to lysed cells, but after a 24-h incubation period, a comparable concentration of thymine was measured in the supernatant compared to lysed cells. This indicates that intact cells expressing TP can phosphorylate thymidine, and thymidine and thymine are able to cross the cell membrane.

A

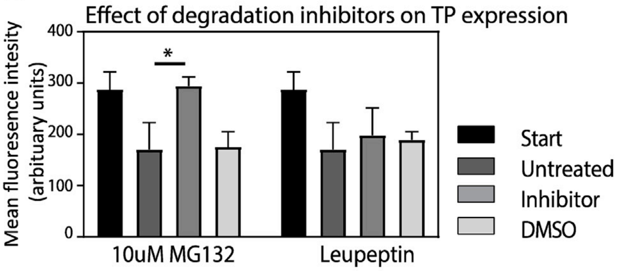

B

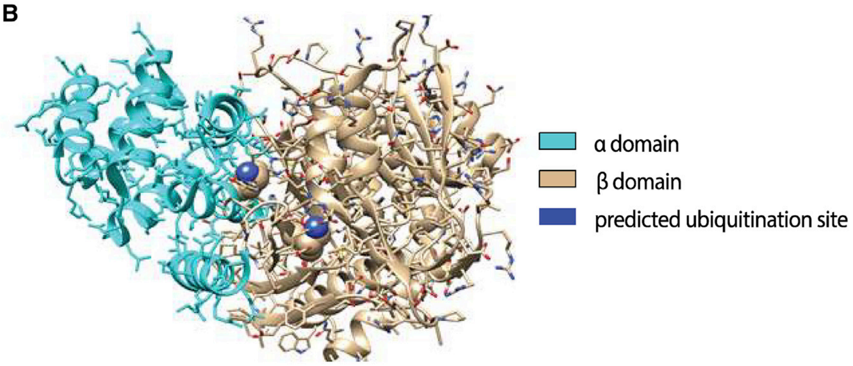

Figure 3. Human TP Is Lost during Differentiation via the Ubiquitination Pathway

To determine the mechanism responsible for TP degradation, 1 × 10^6 day 14 cTP cells (which correspond to the polychromatic erythroblast stage) were incubated with either 5 μM MG132 or 10 μM leupeptin or left untreated at 37°C for 24 h. (A) Graph shows expression levels measured using flow cytometry before addition of the inhibitors (start) and after incubation with the indicated inhibitors or an equivalent concentration of DMSO vehicle as a control (N = 3 ± SEM). (B) Ribbon structure of the human TP enzyme. The α domain is depicted in blue, and the β domain is depicted in beige. The ubiquitination residues that correspond to the known murine ubiquitination sites within the TP active site are labeled in purple.

The cTP cells are morphologically comparable to UT cells by cytospin during differentiation (Figure S2A), and we confirmed by confocal microscopy that TP is localized in the cytoplasm in reticulocytes (Figure S2B). Expression of essential membrane proteins was measured on unmodified control and cTP reticulocytes, and no differences were observed (Figure S2C). The deformability and size of the cTP-derived reticulocytes was measured using an automated rheoscope cell analyzer (ARCA).[20] This showed that the deformability of cTP reticulocytes is comparable to that of the unmodified control reticulocytes and that they have a comparable size (Figure S2D).

TP Is Degraded by the Ubiquitin Degradation Pathway in Erythroblasts

The substantial loss of TP enzyme expression during differentiation suggested to us that TP is likely being actively degraded during terminal differentiation. Ubiquitination and lysosomal degradation are the main cellular degradation pathways during erythropoiesis.[21] To test whether exogenous TP degradation during differentiation is due to ubiquitination or lysosomal degradation, we subjected the day 14 (orthochromatic erythrocytes) cTP cells to the proteasome inhibitor MG132, or a lysosomal degradation inhibitor, leupeptin,[22,23] during culture. TP expression was first measured on day 14 and then 24 h after incubation with MG132, leupeptin, or the vehicle control. Figure 3A shows that application of MG132 inhibited degradation, whereas leupeptin and the vehicle control had no effect. This demonstrates that ubiquitination during erythroid differentiation is a significant cause of human TP protein degradation.

Modeling of Human TP and Mutagenesis of the Ubiquitination Site

Ubiquitination is an essential part of the erythroid cell maturation process,[24] and therefore the global inhibition of ubiquitination-mediated degradation using proteasome inhibitors such as MG132 is not a viable solution to enhancing TP levels in reticulocytes. Examination of the human crystal structure of the TP dimer (protein data bank: 2J0F.pdb) showed that the protein is composed of two homodimers, each consisting of a six-α helix α domain and an α/β domain that consists of an antiparallel β sheet surrounded by α helixes.[25] Interestingly, these domains can rotate 8° relative to each other upon substrate binding.[25] Without substrate present, TP is in an open conformation, while binding to thymidine and phosphate causes the enzyme to close.[25] Kinetic studies conducted on *E. coli* and rabbit TP protein have shown that there is a sequential mechanism of binding whereby the substrate thymidine is the first to bind, and 2-deoxyribose-1-phosphate (2dR1P) is the last to be released.[26]

Human and mouse TP proteins are 81% identical, and sequence comparison confirmed that two known ubiquitination sites located at residues 115 and 221 in the mouse TP enzyme[27] are conserved in the human TP structure. Examination of the structures suggested to us that both conserved lysines are an integral part of the thymidine binding site, and therefore the alteration of these important residues could abolish TP activity or stability because the active site is altered (see Figure 3B). The two lysine residues in human TP were exchanged for arginine residues to preserve the active site structure but remove the ubiquitination sites, making TP-mutant (mut). This enzyme was expressed in both day 3 *in vitro* cultured erythrocytes (cTP-mut) and expanding BEL-A cells (bTP-mut), and the cells subsequently differentiated. The TP-mut expression levels achieved were comparable to day 6 cTP and expanding bTP cells (see Figures 4A and 4B), but no TP activity was detected in the cTP-mut reticulocyte (Figure 4C). This supports our conjecture that the mutations

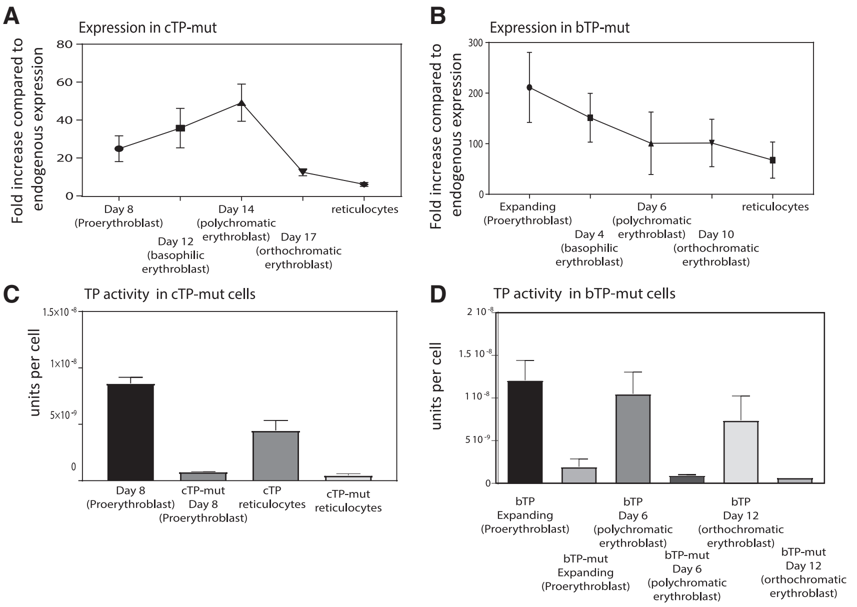

Figure 4. Mutagenesis of the Ubiquitination Sites in the TP Enzyme

(A and B) $CD34^+$ hematopoietic stem cells or expanding BEL-A cells were transduced with TP-mut, a version of the enzyme that had the two mutated ubiquitination sites within the substrate binding domain, generating cTP-mut (A) and bTP-mut (B). The TP-mut overexpressing cells were subsequently differentiated into reticulocytes. TP expression was assessed at different time points during differentiation by flow cytometry as indicated, where 1×10^5 cells were fixed and permeabilized and subsequently stained with a TP antibody. Expression was normalized to endogenous expression of TP in proerythroblasts (N = 5 ± SEM). (C and D) TP activity was measured by a spectrophotometry-based assay in 1×10^6 lysed cTP-mut proerythroblast and reticulocytes (C) and bTP-mut cells during differentiation (D), which shows that the expressed mutant TP enzyme has no activity compared to cTP cells at the same stage of development (N = 5 ± SEM).

compromise the enzyme activity and stability. Therefore, either the enzyme active site would need to be reengineered to retain activity but remove ubiquitin sites, or an alternate means of disrupting TP ubiquitination is required.

TP Degradation Is Reduced by Thymidine Supplementation

After further examination of the molecular human TP structure we observed that the two ubiquitination sites that correspond with murine TP would only be available for ubiquitination in the absence of substrate. We therefore hypothesized that supplementation of the culture media with TP enzyme substrate thymidine would result in a closed TP structure, reducing its degradation by obscuring access to the lysine's ubiquitination sites in the active site. However, previous reports have shown that the addition of thymidine can arrest the cell cycle and also inhibit cell growth in K562 cells at a concentration of 1 mM.[28,29] To determine whether increased thymidine concentrations in our culture media could prevent TP degradation, media were daily supplemented with 0.5 mM thymidine starting at different points during differentiation. We confirmed an increase in cell death and inhibition of differentiation when thymidine was added early during *in vitro* culture, e.g., on day 0 of differentiation (data not shown). To circumvent this, 0.5 mM thymidine supplementation was added daily during differentiation, from day 14 (polychromatic erythroblast stage) in cTP, which is approximately the point when overexpressed human TP is observed to be degraded. A growth curve confirmed that the addition of thymidine from day 14 did not affect cell growth (Figure 5A). This maneuver doubled TP enzyme abundance in cTP reticulocytes (Figure 5B) compared to cells produced using the standard differentiation media, which was not supplemented with thymidine. The enzyme activity assay for the thymidine supplemented cTP was not significantly different from the untreated CTP, and we presume that this is due to the cells already containing excess thymidine before lysis, which distorts the assay. As an additional control we also added a known TP inhibitor (TPI), and we show that this significantly reduces the TP activity (Figure 5C).

DISCUSSION

This study has demonstrated for the first time that exogenous human TP can be overexpressed using lentivirus transduction of the TP cDNA in $CD34^+$ hematopoietic cells and in an erythroid line, BEL-A. These cells were subsequently expanded and differentiated to reticulocytes with confirmed retention of TP. We have also demonstrated that although it is possible to express TP in *in vitro*-cultured reticulocytes, the enzyme is degraded by ubiquitination during differentiation, limiting the abundance of TP per reticulocyte. Ubiquitination is a necessary process during differentiation to remove non-erythroid and non-essential proteins, and therefore this presents a significant barrier to the expression of exogenous proteins for therapeutic purposes during red blood cell development. To circumvent this problem, we propose that a first step for overexpressing proteins is the removal of known or putative ubiquitination sites that are identified in the sequence of exogenous proteins, to prevent loss during degradation. For enzymes where the ubiquitination site is an integral part of the substrate binding site such as in the case of TP, an alternative strategy is needed, as an alteration of the ubiquitination motif within the active site can disrupt activity. Our novel observation shown here for TP is that inclusion of the TP enzyme substrate thymidine increases the abundance of the TP in reticulocytes, likely by masking the enzyme ubiquitin sites. Although thymidine is known to be toxic to cells, we show that this can be achieved by supplementing the culture medium with substrate (in this case thymidine) during the late stages of terminal differentiation. Furthermore, since patients have a concentration of >3 μmol/L of thymidine in their blood,

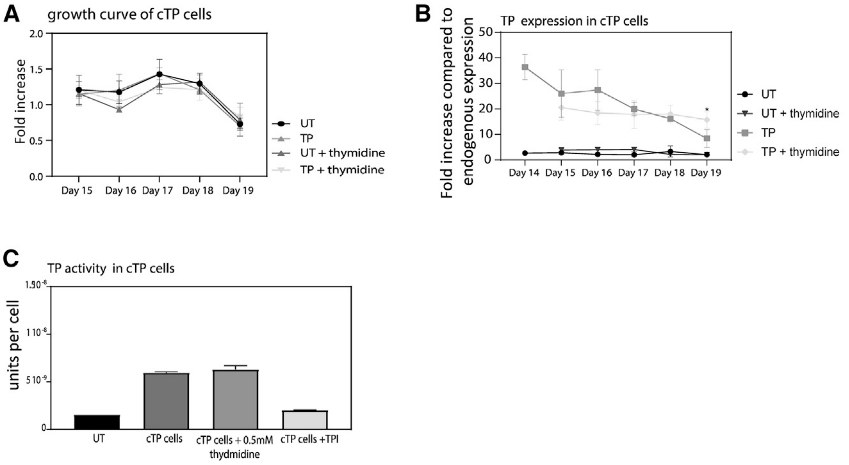

Figure 5. TP Expression Is Significantly Increased by Supplementing the Culturing Media with Thymidine

The expression TP in cTP and cells during differentiation was increased by supplementing the culturing media with 0.5 mM thymidine every 24 h from day 14 (polychromatic stage) in cTP cells. (A) Growth curve shows the fold increase in cell number during differentiation with the absence or addition of 0.5 mM thymidine. (B) Expression of TP was measured by flow cytometry using 1×10^5 fixed and permeabilized reticulocytes from cultures either untreated in standard media or treated with media supplemented by thymidine (N = 4 ± SEM). (C) Specific TP activity was confirmed by an activity assay where we used a thymidine phosphorylase inhibitor (TPI) as a negative control.

this should maintain TP in reticulocytes while they mature to red blood cells.

A clinical trial in a MNGIE patient conducted by Bax and colleagues[30] successfully encapsulated a therapeutic dose of 51 U of *E. coli*-produced TP in 10^{10} erythrocytes, whereby 1 U of TP is defined as the activity required to convert 1.0 μmol of thymidine to thymine per minute. Our cTP-cultured erythroblasts had an average expression of TP corresponding to 84 U in 10^{10} cells. The expression level decreases during differentiation and results in an activity corresponding to 44 U in 10^{10} reticulocytes. We show that TP expression can be doubled in reticulocytes by daily addition of thymidine in the culture medium during the late stages of differentiation. Although the addition of thymidine did prevent degradation, we acknowledge that TP will still be partially lost in the cytosol surrounding the nucleus during enucleation, and some loss may also occur during the process of reticulocyte maturation when cells reduce in size.[31]

The advantage of the use of *in vitro*-cultured reticulocytes modified genetically to express and retain TP is that these cells overproduce the enzyme during normal differentiation and have not been subjected to a damaging hypotonic lysis, which is required for the encapsulation of TP in red blood cells. In addition, since the cultured reticulocytes are newly synthesized nascent red blood cells, they are expected to have a normal red blood cell lifespan of up to 120 days, potentially reducing the number of transfusions needed for patients compared to using donated blood derived red blood cells that is a mixture of ages.

The utility of *in vivo* animal models to assess survival and efficacy of transfused human red blood cells is limited by the short time frame (48–72 h) in which these cells are able to circulate, even where immunocompromised (macrophage-depleted NSG mice) mouse models have been employed,[16] and although a mouse model that recapitulates the MNGIE phenotype has been previously described,[32] such a model does not exist on a NSG background. As such, there is currently no way to pre-assess the survival or efficiency of excess thymidine removal from sera by modified red blood cells in circulation. Nevertheless, data derived from a single injection using autologous *in vitro*-cultured reticulocytes suggest that these cultured reticulocytes have a half-life of 28 ± 2 days, which was comparable to a normal red blood cell transfusion.[33] Therefore, the use of modified *in vitro*-cultured reticulocytes with TP could decrease the frequency of transfusions needed, which would reduce the risk of transfusion-related symptoms. Another advantage of using *in vitro*-cultured TP reticulocytes is that we have used unmodified human TP, which is unlikely to induce an immune response.

In summary, this study has provided the first proof of principle demonstration of the enhancement of *ex vivo*-produced reticulocytes, from either CD34$^+$ cells or an erythroid line, by engineering the cells during red cell development to overexpress TP as a novel cellular therapy treatment for MNGIE. The CD34$^+$-derived engineered reticulocytes could be made autologous or allogeneic, and because these are red blood cells that have lost their nuclei, there is no anticipated regulatory barrier to using lentivirus on the cells. Given that GMP-compliant production of reticulocytes is now feasible for small volumes in a process amenable to modification by overexpressing enzymes, and a proof-of-principle autologous transfusion into a volunteer has been conducted and further allogenic trials are planned,[34,35] the enhancement of reticulocyte function through lentivirus transduction of erythroid precursors may represent an early therapeutic use of *in vitro*-cultured blood in the clinic. We acknowledge that although the culture efficiency of the BEL-A cells and scalability need to be improved and a suitable GMP-compatible erythroid line needs to be produced, the production of erythroid lines containing overexpressed therapeutic proteins represents a more convenient and sustainable way to make a future therapeutic reticulocyte product for patients.[36] Furthermore, when combined with gene editing approaches using CRISPR-Cas9, these cell lines can be enhanced further to produce an engineered therapeutic product designed to be compatible to a broader range of patients.[36]

MATERIALS AND METHODS

Antibodies

Monoclonal TP antibody (clone P-GF.44C) was used at 1:10 dilution (Thermo Scientific, Gillingham, UK). For membrane protein analysis, 1:1 (v/v) diluted GPC (BRIC4), CD47 (BRIC32), Rh (BRIC69), Band3 (BRIC71), GPA (BRIC256), and RhAG (LA1818) (IBGRL Reagents, Bristol, UK) were used. Secondary antibodies used were allophycocyanin (APC)-conjugated monoclonal anti-mouse immunoglobulin G (IgG)1 or polyclonal anti-IgG (BioLegend, London, UK) or Alexa Fluor 647-anti-human (Jackson ImmunoResearch Laboratories, Cambridgeshire, UK) and used at 1:50 (v/v). For confocal imaging, a polyclonal GPC C-terminal antibody at 1:50 was used (in-house). Secondary antibodies used were goat anti-rabbit-Alexa Fluor 594 (Invitrogen, Massachusets, USA) and goat anti-mouse-Alexa Fluor 488 (Invitrogen).

BEL-A Cell Culture

BEL-A cells were cultured as previously described.[17] In brief, cells were maintained in expansion medium (StemSpan serum-free expansion medium [SFEM; STEMCELL Technologies, Cambridge, UK] supplemented with 50 ng/mL stem cell factor [SCF; Miltenyi Biotec, Bisley, UK], 3 U/mL erythropoietin [EPO; Roche, Welwyn Garden City, UK], 1 μM dexamethasone [Sigma-Aldrich, Poole, UK], and 1 μg/mL doxycycline [Sigma-Aldrich]) at 1–3 × 10^5 cells/mL. Complete medium changes were performed every 48 h. Differentiation was induced as previously described: cells were seeded at 1.5 × 10^5/mL in differentiation medium (Iscove's modified Dulbecco's medium [IMDM], Source BioScience, Nottingham, UK) containing 3% (v/v) AB serum (Sigma-Aldrich), 2 mg/mL human serum albumin (HSA) (Irvine Scientific, Newtown Mount Kennedy, Ireland), 10 μg/mL insulin (Sigma-Aldrich), 3 U/mL heparin (Sigma-Aldrich), 500 μg/mL transferrin (Sanquin Blood Supply, the Netherlands), and 3 U/mL EPO (Roche, Welwyn Garden City, UK) supplemented with 1 ng/mL interleukin (IL)-3 (R&D Systems, Abingdon, UK), 10 ng/mL SCF, and 1 μg/mL doxycycline. After 2 days, cells were reseeded at 3 × 10^5/mL in fresh medium. On differentiation day 4, cells were reseeded at 5 × 10^5/mL in fresh medium without doxycycline. On differentiation day 6, a complete media change was performed, and cells were reseeded at 1 × 10^6/mL. On day 8, cells were transferred to differentiation medium (containing no SCF, IL-3, or doxycycline) and maintained at 1 × 10^6/mL with complete medium changes every 2 days until day 12.[17,36]

CD34$^+$ Cell Culture

The donated blood from apheresis waste was provided with written informed consent for research use given in accordance with the Declaration of Helsinki from National Health Service Blood and Transplant, Filton, Bristol, United Kingdom). The studies on erythropoiesis and research protocols were reviewed and approved by the Bristol Research Ethics committee (REC Number 12/SW/0199). As previously described,[19] CD34$^+$ hematopoietic stem cells were isolated from human blood donor mononuclear cells or from thawed cryopreserved cord blood units by magnetic bead separation according to the manufacturer's instructions (Miltenyi Biotec). CD34$^+$ cells were grown at a density of 2 × 10^5 cells/mL using a base medium consisting of IMDM (Source BioScience) containing 3% (v/v) AB serum, 2 mg/mL HSA, 10 μg/mL insulin, 3 U/mL heparin, 500 μg/mL transferrin, and 3 U/mL erythropoietin. In the first stage (days 0–10) this was supplemented with 10 ng/mL SCF and 1 ng/mL IL-3, and in the second stage (days 11–13) with 10 ng/mL SCF. In the final stage to day 19, only the base medium was used. 10 μM leupeptin (Sigma-Aldrich) or 5 μM MG132 (Sigma-Aldrich) or vehicle was added at day 14 to 1 × 10^6 differentiating erythroblasts for 24 h at 37°C, 5% CO_2.

Lentiviral Transduction

A human TP cDNA sequence was ordered and cloned in the XLG3 vector by GenScript (Leiden, the Netherlands). The original TP sequence was mutated at sites 115 and 221 from a lysine to arginine, thereby creating the TP-mut lentivirus as prepared according to previously published protocols.[37] For transduction of BEL-A and CD34$^+$ hematopoietic cells, virus was added to 2 × 10^5 cells in 2 mL of medium in the presence of 8 μg/mL Polybrene for 24 h. Cells were washed three times and resuspended in fresh medium.

Flow Cytometry and FACS

For flow cytometry on undifferentiated BEL-A cells, 1 × 10^5 cells were fixed in 1% paraformaldehyde and 0.0075% glutaraldehyde, permeabilized with 0.1% Triton X-100, resuspended in PBSAG (PBS + 1 mg/mL bovine serum albumin [BSA], 2 mg/mL glucose) + 1% BSA, and labeled with primary antibody for 30 min at 4°C. Cells were washed in PBSAG, incubated for 30 min at 4°C with appropriate APC-conjugated secondary antibody, and washed, and data were acquired on a MACSQuant VYB analyzer using a plate reader. Reticulocytes were identified by gating on a Hoechst-negative population. For FACS sorting of cells, a BD Influx cell sorter was used to isolate single clones by sorting the propidium iodide-negative population into 96-well plates.

TP Activity Assay

1 × 10^6 cells were resuspended in lysis buffer (50 mM Tris-HCl [pH 7.2], 1% [w/v] Triton X-100, 2 mM phenylmethylsulfonyl fluoride [PMSF], 0.02% [v/v] 2-mercaptoethanol). The lysate was centrifuged at 16,000 × g for 30 min at 4°C and then 176 mM thymidine and 5× TP reaction buffer (0.5 M Tris-arsenate [pH 6.5]) was added to the supernatant. The controls used were lysis buffer alone or lysis buffer containing known concentrations of purified TP protein (Sigma-Aldrich). The reaction was incubated at 37°C for 60 min and then terminated by addition of 0.3 M NaOH. The absorbance was measured using a spectrophotometer at 299 nm and compared to the TP enzyme standard concentration curve.[38]

Cytospin Staining

1 × 10^5 cells were cytospun onto glass slides, fixed in methanol, and stained with May-Grünwald-Giemsa stains according to the manufacturer's protocol. Images were taken with a Leica DM750

microscope coupled to an Olympus U-TVO.5XC-3 camera using a ×40 lens and processed using Adobe Photoshop 9.0 (Adobe Systems).

Immunofluorescence

5×10^5 cells were fixed in suspension in 1% paraformaldehyde and 0.0075% glutaraldehyde and washed three times in PBSAG before being cytospun onto coated coverslips. Cells were then permeabilized with 0.05% Triton X-100 for 5 min at room temperature and then blocked in PBS-4% BSA for 45 min, incubated with primary antibodies in PBS-4% BSA for 1 h, washed with PBS, and incubated for 1 h with the appropriate secondary antibodies. Coverslips were washed and mounted on microscope slides using Mowiol (Calbiochem, San Diego, USA) containing 2.5% (w/v) DABCO antifade reagent (Sigma-Aldrich). Confocal images were taken using a Leica SPE single-channel confocal laser scanning microscope attached to a Leica DMi8 inverted epifluorescence microscope.

SDS-PAGE and Western Blotting

1×10^6 cells were taken at designated time points during differentiation and pelleted, snap-frozen, and stored at −80°C. Pellets were lysed for 10 min on ice in lysis buffer (20 mM Tris-HCl [pH 8.0], 137 mM NaCl, 10 mM EDTA, 100 mM NaF, 1% [v/v] Nonidet P-40, 10% [v/v] glycerol, 10 mM Na_3VO_4, 2 mM PMSF, and protease inhibitors [Calbiochem]). Equal numbers of lysed cells were loaded and separated by SDS-PAGE and then immunoblotted.

Reticulocyte Deformability Measurements Using ARCA

1×10^6 reticulocytes were resuspended in 200 μL of polyvinylpyrrolidone (PVP) solution (viscosity, 28.1; Mechatronics Instruments, the Netherlands). Samples were assayed in an ARCA (automated rheoscope and cell analyser) assay,[39] which consists of a plate-to-plate optical shearing stage (model CSS450) mounted on a Linkam imaging station assembly and temperature controlled using Linksys32 software (Linkam Scientific Instruments, Surrey, UK) at 37°C with 3 Pa. The microscope was equipped with an LMPlanFl 50× objective lens with a 10.6-mm working distance objective (Olympus, Essex, UK) illuminated by an X-1500 stroboscope (PerkinElmer, the Netherlands) through a band-pass interference filter (center wavelength [CWL], 420 nm; full width at half maximum [FWHM], 10 nm; Edmund Optics, Poppleton, UK). Images were acquired using a uEye camera (UI-2140SE-M-GL; IDS, Obersulm, Germany). At least 1,000 cell images per sample were acquired and analyzed using bespoke ARCA software. In this analysis the distribution of the surface area of the red blood cells within the sample as observed in the ARCA assay was estimated as well as the distribution of the degree of deformation of the red blood cells due to the imposed shear stress.

TP Modeling

Ubiquitination sites were predicted from mUbiSiDa, a database of mammalian protein ubiquitination sites (https://omictools.com/mubisida-tool), which provided details of the mouse ubiquitination sites analyzed in Wagner et al.[27] Clustal Omega (http://www.clustal.org/omega/) was used to align human and mouse sequences. Protein structures were visualized, and images were produced with UCSF Chimera software (https://www.cgl.ucsf.edu/chimera/).

SUPPLEMENTAL INFORMATION

Supplemental Information can be found online at https://doi.org/10.1016/j.omtm.2020.03.029.

AUTHOR CONTRIBUTIONS

Experiments were conceived and designed by M.M. and A.M.T. M.M. and D.S. carried out the experiments, performed the analysis and prepared the figures. J.F. provided the BEL-A cell line. J.G.G.D., G.J.S. and J.F. edited the manuscript. A.M.T. supervised the project and edited the manuscript. All authors read and approved the manuscript.

ACKNOWLEDGMENTS

We thank Dr. Tim Satchwell for critical reading of the manuscript. We also thank Sanquin Blood Supply (the Netherlands) for the supply of human transferrin. This work was funded by BrisSynBio via the BBSRC/EPSRC Synthetic Biology Research Centre (BB/L01386X/1) and also the Defence Science and Technology Laboratory (DSTL), along with funding from NHS Blood and Transplant (NHSBT) R&D (WP15-05) and NIHR Blood and Transplant Research Unit (NIHR BTRU) in Red Cell Products (IS-BTU-1214-10032). This manuscript presents independent research funded in part by the National Institute for Health Research (NIHR). The views expressed are those of the authors and not necessarily those of the NHS, the NIHR, or the Department of Health and Social Care.

REFERENCES

1. Okamura, K., Santa, T., Nagae, K., and Omae, T. (1976). Congenital oculoskeletal myopathy with abnormal muscle and liver mitochondria. J. Neurol. Sci. *27*, 79–91.
2. Garone, C., Tadesse, S., and Hirano, M. (2011). Clinical and genetic spectrum of mitochondrial neurogastrointestinal encephalomyopathy. Brain *134*, 3326–3332.
3. Hirano, M., Nishigaki, Y., and Martí, R. (2004). Mitochondrial neurogastrointestinal encephalomyopathy (MNGIE): a disease of two genomes. Neurologist *10*, 8–17.
4. Yadak, R., Sillevis Smitt, P., van Gisbergen, M.W., van Til, N.P., and de Coo, I.F. (2017). Mitochondrial neurogastrointestinal encephalomyopathy caused by thymidine phosphorylase enzyme deficiency: from pathogenesis to emerging therapeutic options. Front. Cell. Neurosci. *11*, 31.
5. DiMauro, S., and Schon, E.A. (2003). Mitochondrial respiratory-chain diseases. N. Engl. J. Med. *348*, 2656–2668.
6. Nishino, I., Spinazzola, A., and Hirano, M. (1999). Thymidine phosphorylase gene mutations in MNGIE, a human mitochondrial disorder. Science *283*, 689–692.
7. Spinazzola, A., Marti, R., Nishino, I., Andreu, A.L., Naini, A., Tadesse, S., Pela, I., Zammarchi, E., Donati, M.A., Oliver, J.A., and Hirano, M. (2002). Altered thymidine metabolism due to defects of thymidine phosphorylase. J. Biol. Chem. *277*, 4128–4133.
8. Röeben, B., Marquetand, J., Bender, B., Billing, H., Haack, T.B., Sanchez-Albisua, I., Schöls, L., Blom, H.J., and Synofzik, M. (2017). Hemodialysis in MNGIE transiently reduces serum and urine levels of thymidine and deoxyuridine, but not CSF levels and neurological function. Orphanet J. Rare Dis. *12*, 135.
9. Young, M.J., and Copeland, W.C. (2016). Human mitochondrial DNA replication machinery and disease. Curr. Opin. Genet. Dev. *38*, 52–62.
10. Peedikayil, M.C., Kagevi, E.I., Abufarhaneh, E., Alsayed, M.D., and Alzahrani, H.A. (2015). Mitochondrial neurogastrointestinal encephalomyopathy treated with stem cell transplantation: a case report and review of literature. Hematol. Oncol. Stem Cell Ther. *8*, 85–90.

11. Ihler, G.M., Glew, R.H., and Schnure, F.W. (1973). Enzyme loading of erythrocytes. Proc. Natl. Acad. Sci. USA *70*, 2663–2666.

12. Bourgeaux, V., Lanao, J.M., Bax, B.E., and Godfrin, Y. (2016). Drug-loaded erythrocytes: on the road toward marketing approval. Drug Des. Devel. Ther. *10*, 665–676.

13. Bax, B.E., Bain, M.D., Scarpelli, M., Filosto, M., Tonin, P., and Moran, N. (2013). Clinical and biochemical improvements in a patient with MNGIE following enzyme replacement. Neurology *81*, 1269–1271.

14. Bax, B.E., Levene, M., Bain, M.D., Fairbanks, L.D., Filosto, M., Kalkan Uçar, S., Klopstock, T., Kornblum, C., Mandel, H., Rahman, S., et al. (2019). Erythrocyte encapsulated thymidine phosphorylase for the treatment of patients with mitochondrial neurogastrointestinal encephalomyopathy: study protocol for a multi-centre, multiple dose, open label trial. J. Clin. Med. *8*, E1096.

15. Levene, M., Pacitti, D., Gasson, C., Hall, J., Sellos-Moura, M., and Bax, B.E. (2018). Validation of an immunoassay for anti-thymidine phosphorylase antibodies in patients with MNGIE treated with enzyme replacement therapy. Mol. Ther. Methods Clin. Dev. *11*, 1–8.

16. Kupzig, S., Parsons, S.F., Curnow, E., Anstee, D.J., and Blair, A. (2017). Superior survival of *ex vivo* cultured human reticulocytes following transfusion into mice. Haematologica *102*, 476–483.

17. Trakarnsanga, K., Griffiths, R.E., Wilson, M.C., Blair, A., Satchwell, T.J., Meinders, M., Cogan, N., Kupzig, S., Kurita, R., Nakamura, Y., et al. (2017). An immortalized adult human erythroid line facilitates sustainable and scalable generation of functional red cells. Nat. Commun. *8*, 14750.

18. Friedkin, M., and Roberts, D. (1954). The enzymatic synthesis of nucleosides. I. Thymidine phosphorylase in mammalian tissue. J. Biol. Chem. *207*, 245–256.

19. Griffiths, R.E., Kupzig, S., Cogan, N., Mankelow, T.J., Betin, V.M., Trakarnsanga, K., Massey, E.J., Lane, J.D., Parsons, S.F., and Anstee, D.J. (2012). Maturing reticulocytes internalize plasma membrane in glycophorin A-containing vesicles that fuse with autophagosomes before exocytosis. Blood *119*, 6296–6306.

20. Dobbe, J.G., Streekstra, G.J., Hardeman, M.R., Ince, C., and Grimbergen, C.A. (2002). Measurement of the distribution of red blood cell deformability using an automated rheoscope. Cytometry *50*, 313–325.

21. Ciechanover, A. (2006). Intracellular protein degradation: from a vague idea thru the lysosome and the ubiquitin-proteasome system and onto human diseases and drug targeting. Exp. Biol. Med. (Maywood) *231*, 1197–1211.

22. Tsubuki, S., Saito, Y., Tomioka, M., Ito, H., and Kawashima, S. (1996). Differential inhibition of calpain and proteasome activities by peptidyl aldehydes of di-leucine and tri-leucine. J. Biochem. *119*, 572–576.

23. Hershko, A., and Ciechanover, A. (1982). Mechanisms of intracellular protein breakdown. Annu. Rev. Biochem. *51*, 335–364.

24. Nguyen, A.T., Prado, M.A., Schmidt, P.J., Sendamarai, A.K., Wilson-Grady, J.T., Min, M., Campagna, D.R., Tian, G., Shi, Y., Dederer, V., et al. (2017). UBE2O remodels the proteome during terminal erythroid differentiation. Science *357*, eaan0218.

25. Norman, R.A., Barry, S.T., Bate, M., Breed, J., Colls, J.G., Ernill, R.J., Luke, R.W., Minshull, C.A., McAlister, M.S., McCall, E.J., et al. (2004). Crystal structure of human thymidine phosphorylase in complex with a small molecule inhibitor. Structure *12*, 75–84.

26. Krenitsky, T.A. (1968). Pentosyl transfer mechanisms of the mammalian nucleoside phosphorylases. J. Biol. Chem. *243*, 2871–2875.

27. Wagner, S.A., Beli, P., Weinert, B.T., Schölz, C., Kelstrup, C.D., Young, C., Nielsen, M.L., Olsen, J.V., Brakebusch, C., and Choudhary, C. (2012). Proteomic analyses reveal divergent ubiquitylation site patterns in murine tissues. Mol. Cell. Proteomics *11*, 1578–1585.

28. Anisimov, A.G., Chekmasova, A.A., Volkova, T.O., and Nemova, N.N. (2003). [Erythroid differentiation of K562 cells resistant to 2-(4′-dimethylaminostyryl)quinoline 1-oxide or 4-nitroquinoline 1-oxide is significantly increased after thymidine treatment]. Izv. Akad. Nauk Ser. Biol. *2003*, 275–284.

29. Thomas, D.B., and Lingwood, C.A. (1975). A model of cell cycle control: effects of thymidine on synchronous cell cultures. Cell *5*, 37–42.

30. Moran, N.F., Bain, M.D., Muqit, M.M., and Bax, B.E. (2008). Carrier erythrocyte entrapped thymidine phosphorylase therapy for MNGIE. Neurology *71*, 686–688.

31. Bell, A.J., Satchwell, T.J., Heesom, K.J., Hawley, B.R., Kupzig, S., Hazell, M., Mushens, R., Herman, A., and Toye, A.M. (2013). Protein distribution during human erythroblast enucleation in vitro. PLoS ONE *8*, e60300.

32. Yadak, R., Cabrera-Pérez, R., Torres-Torronteras, J., Bugiani, M., Haeck, J.C., Huston, M.W., Bogaerts, E., Goffart, S., Jacobs, E.H., Stok, M., et al. (2018). Preclinical efficacy and safety evaluation of hematopoietic stem cell gene therapy in a mouse model of MNGIE. Mol. Ther. Methods Clin. Dev. *8*, 152–165.

33. Douay, L. (2012). In vitro generation of red blood cells for transfusion: a model for regenerative medicine. Regen. Med *7*, 1–2.

34. Anstee, D.J., Gampel, A., and Toye, A.M. (2012). Ex-vivo generation of human red cells for transfusion. Curr. Opin. Hematol. *19*, 163–169.

35. van den Akker, E., Satchwell, T.J., Pellegrin, S., Daniels, G., and Toye, A.M. (2010). The majority of the in vitro erythroid expansion potential resides in $CD34^{-}$ cells, outweighing the contribution of $CD34^{+}$ cells and significantly increasing the erythroblast yield from peripheral blood samples. Haematologica *95*, 1594–1598.

36. Hawksworth, J., Satchwell, T.J., Meinders, M., Daniels, D.E., Regan, F., Thornton, N.M., Wilson, M.C., Dobbe, J.G., Streekstra, G.J., Trakarnsanga, K., et al. (2018). Enhancement of red blood cell transfusion compatibility using CRISPR-mediated erythroblast gene editing. EMBO Mol. Med. *10*, e8454.

37. Satchwell, T.J., Bell, A.J., and Toye, A.M. (2015). The sorting of blood group active proteins during enucleation. ISBT Sci. Ser. *10* (*Suppl 1*), 163–168.

38. Martí, R., López, L.C., and Hirano, M. (2012). Assessment of thymidine phosphorylase function: measurement of plasma thymidine (and deoxyuridine) and thymidine phosphorylase activity. Methods Mol. Biol. *837*, 121–133.

39. Dobbe, J.G., Streekstra, G.J., Hardeman, M.R., Ince, C., and Grimbergen, C.A. (2002). Measurement of the distribution of red blood cell deformability using an automated rheoscope. Cytometry *50*, 313–325.

OMTM, Volume *17*

Supplemental Information

Expression and Retention of Thymidine Phosphorylase in Cultured Reticulocytes as a Novel Treatment for MNGIE

Marjolein Meinders, Debbie Shoemark, Johannes G.G. Dobbe, Geert J. Streekstra, Jan Frayne, and Ashley M. Toye

Supplemental Figure 1

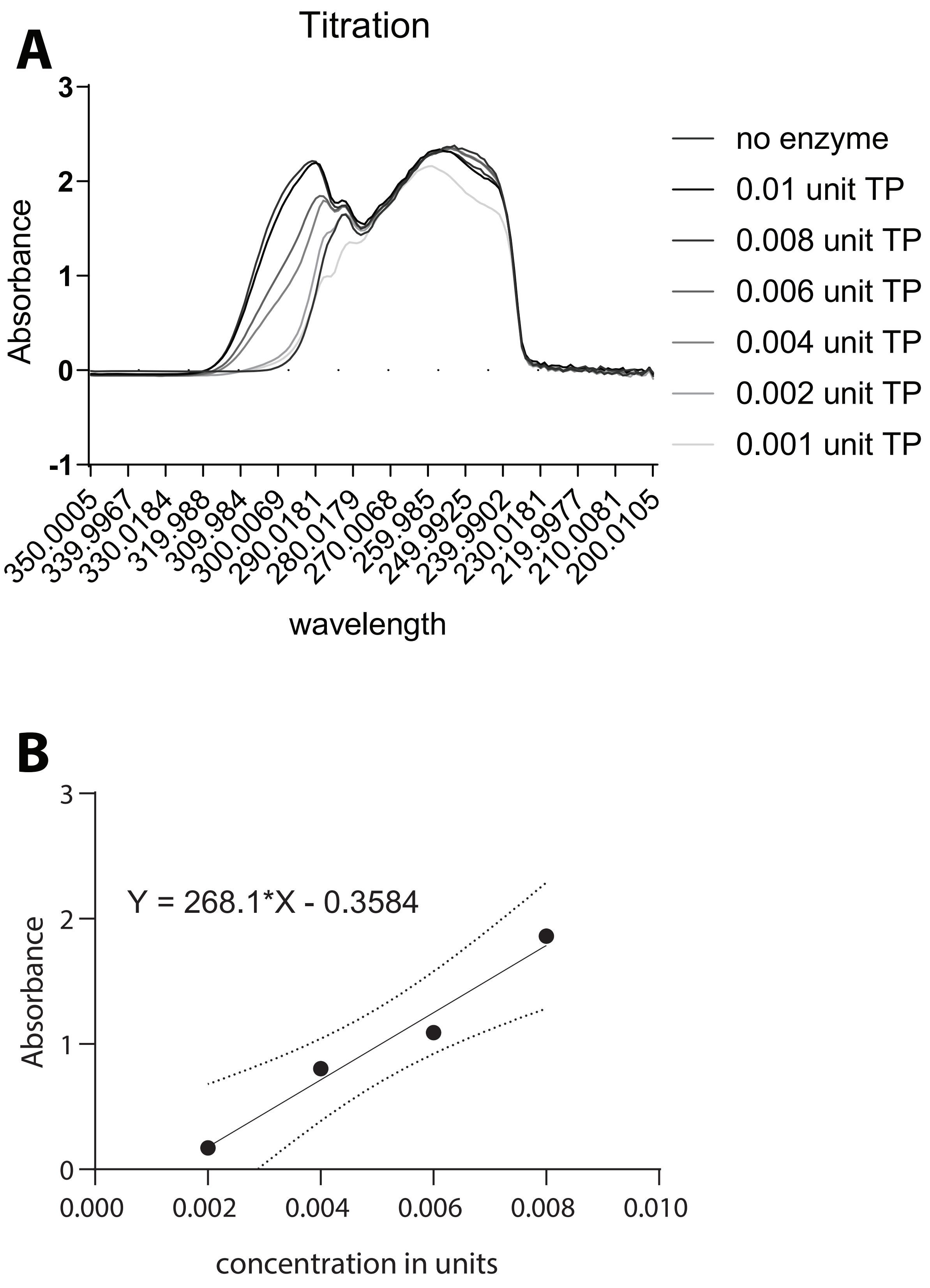

Supplemental Figure 2

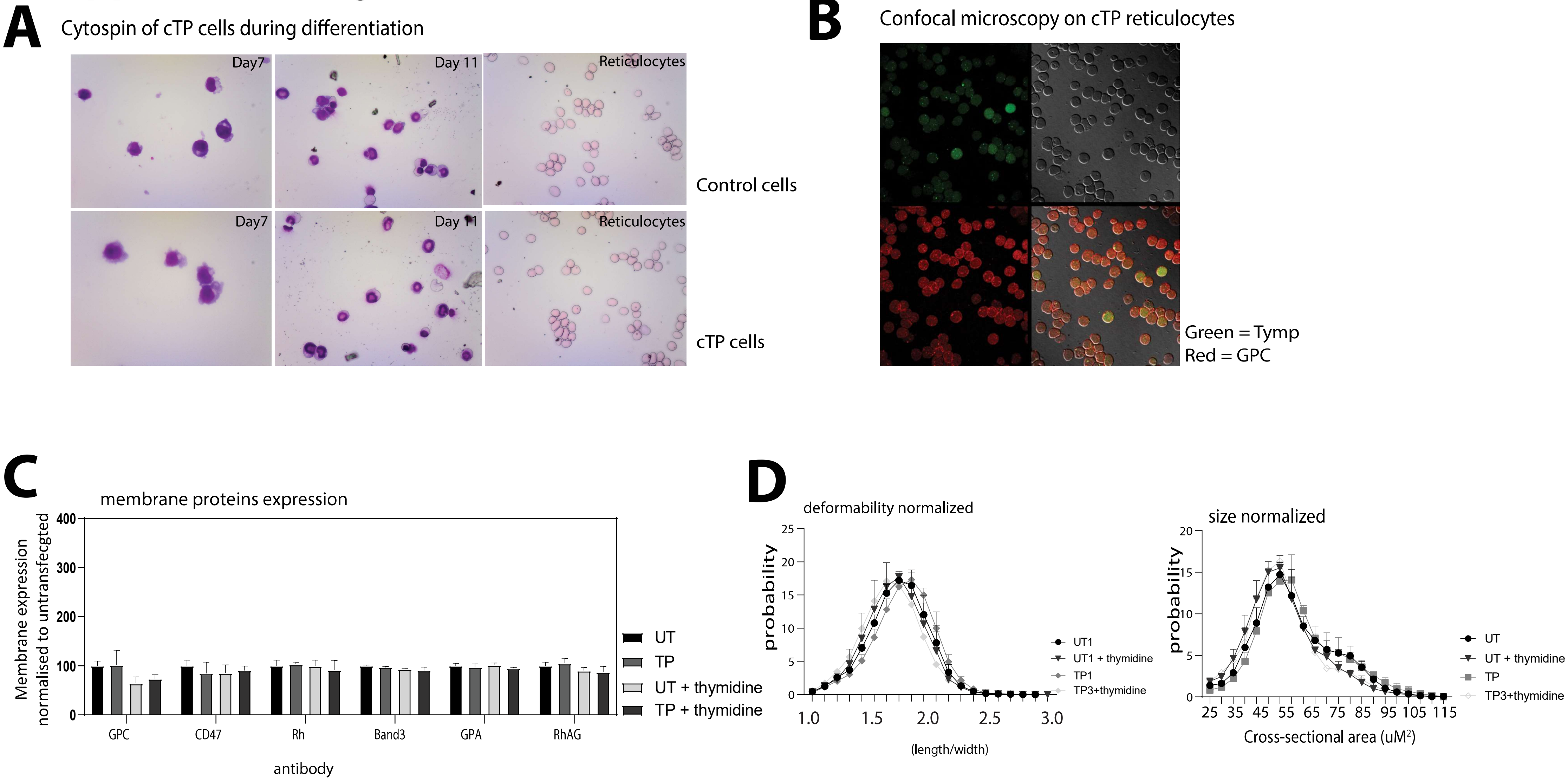

Supplementary Figure 1. TP activity assay data acquisition and calculation

For each individual experiment a standard curve was done to calculate the concentration of TP in our cells in units/cells. An example of the calculation is shown in Supplemental Figure 1. (A) shows the reduction of thymidine upon incubation with a known amount of enzyme after 1-hour incubation at 37°C. (B) shows the absolute absorbance values at wavelength of 299nm. We are able to fit a regression line through the data points from which we can calculate the formula y=268.1X-0.3584, whereby X is the concentration in the cell and Y the absorbance. The concentration of TP is in units, which is defined as 1U (μmol/min) is the amount of the enzyme that catalyses the conversion of one micromole of thymidine per minute. This is an example calculation. For each individual experiment, a regression line was plotted, and the concentration was calculated.

Supplementary Figure 2. Additional analysis of cTP reticulocytes

(A) Representative images illustrating cell morphology in UT and cTP cells during differentiation. At indicated times of cell culture, $1x10^4$ cells were cytospun and stained with a may-grunwald giemsa stain. (B) Reticulocytes from cTP were fixed and labelled with a TP (green) and a GPC (red) antibody and imaged using confocal microscopy which confirmed the cytosolic localization of TP in the reticulocytes. (C) Membrane protein expression was measured using flow cytometry on reticulocytes of GPC, CD47, Rh, Band3, GPA, RhAG from untransduced (UT) or untransduced cultured with 0.5mM thymidine, cTP, and cTP cells cultured with thymidine (N=3 ± SEM). (D) Automated Rheoscope and Cell Analyzer (ARCA) measurements of deformability and size for UT (untransduced cells) and cTP reticulocytes cultured in the presence of 0.5mM thymidine (n=3 ± SEM).
